# Specific emitter identification based on multiple sequence feature learning

**DOI:** 10.1371/journal.pone.0299664

**Published:** 2024-05-15

**Authors:** Dong Yi, Di Wu, Tao Hu, Zhifu Tian, Yanyun Wang

**Affiliations:** The School of Data and Target Engineering, PLA Strategic Support Force Information Engineering University, Zhengzhou, China; National Yang Ming Chiao Tung University, TAIWAN

## Abstract

The specific emitter identification is widely used in electronic countermeasures, spectrum control, wireless network security and other civil and military fields. In response to the problems that the traditional specific emitter identification algorithm relies on a priori knowledge and has poor generalizability, and the existing specific emitter identification algorithm based on deep learning has poor feature selection and the adopted feature extraction network is not targeted, etc., the specific emitter identification algorithm based on multi-sequence feature learning is proposed. Firstly, multiple sequence features of the emitted signal of the communication radiation source are extracted, and these features are combined into multiple sequence features. Secondly, the multiple sequence fusion convolutional network is constructed to fuse and deeply extract the multiple sequence features and complete the classification of individual communication radiation sources through the classifier of neural network. The selected sequence features of this algorithm contain more and more essential RFF information, while the targeted design of the multi-sequence feature fusion learning network can effectively extract the essential RFF information. The results show that the algorithm can significantly improve the performance of SEI compared with the benchmark algorithm, with a recognition rate gain of about 17%.

## Introduction

Specific Emitter Identification (SEI) [1], i.e., the unique identification of the target radiation source is achieved by extracting the Radio Frequency Fingerprint (RFF) features on the received signal that can reflect the individual differences of the radiation source [2]. In the field of electronic countermeasures reconnaissance, the ability to accurately and quickly intercept and identify the enemy’s communication signals can provide commanders with more basis for decision making [3]. In the civil field, SEI can be used to identify illegal incoming radio stations, thus securing the communication network and has some academic research value [4].

SEI algorithms can be broadly classified into two categories at present: traditional SEI algorithms and deep learning-based SEI algorithms. Traditional SEI algorithms, in terms of the types of extracted features, can be divided into three categories: signal parameter statistical features [5, 6], signal transform domain statistical features [7, 8], and mechanism model features [9, 10]. These three types of features have achieved fruitful results in different stages and scenarios, but the common problem is that they are limited to human knowledge of signal essence, mathematical tools and radiation source mechanism, and it is more difficult to understand and extract complex essential features to meet the generality, stability and comprehensiveness requirements of SEI.

In recent years, deep learning has been widely developed and applied in the field of SEI [11], and deep neural networks are able to retrieve abstract features through multiple implicit layers with nonlinear activation functions, which is beneficial for extracting deep subtle features of radiation source signals [12]. Yiwei Pan et al. proposed a vector map-based SEI algorithm, which applied deep learning techniques to achieve the joint extraction of multiple complex features [13]. Jinkai Xu et al. designed a SEI algorithm based on variational modal decomposition, and designed deep convolutional neural networks for feature extraction and classification recognition of the decomposed high-frequency components [14]. Jian Wang. et al. extracted multiple transform domain features of the emitted signal from the communication radiation source and combined these features into multi-domain features, after which they constructed a multi-channel convolutional neural network and used multi-channel convolutional operation for deep extraction of multi-domain features. The classification of individual communication radiation sources was accomplished by the classifier of the neural network [15]. He Zunwen et al. proposed a fusion classification algorithm based on multichannel transform projection, integrated deep learning and generative adversarial networks for the SEI problem [16].

The above SEI algorithms either use a single feature or a multi-feature fusion algorithm, and all of them have achieved good results. But the above algorithms have three problems: first, some SEI algorithms use a single feature, and recognition algorithms using only one transform domain information have different recognition performance in different scenes, different channels and noise conditions, and there is no guarantee that artificially selecting a domain feature is the optimal feature, so the phenomenon of sub-optimal recognition effect often occurs. Second, although some SEI algorithms utilize multiple features, they do not design a more effective feature extraction network for multiple feature inputs, resulting in the fusion effect of features and the extraction of RFF information not being optimal. Third, most of the SEI algorithms perform certain mathematical operations on the original signal waveform and turn it into other graphic features or sequence features before extracting fingerprint features. However, the RFF features of the signal are mainly embedded in the sequence waveform, and the mathematical operation to other features, such as graphic features, can make the fingerprint features visually distinguishable, but with the process of mathematical operation of the signal, the RFF features of the signal will inevitably be lost with the operation process, so that the deep network cannot fully utilize the RFF features covered in the signal features.

To solve the above problems, the algorithm in this study takes time-domain in-phase/quadrature (I/Q) sequence features as the main features and supplements time-domain amplitude/phase (A/P) sequence features and frequency-domain A/P sequence features composed as multiple sequences to avoid the problems of unsatisfactory recognition results due to poor feature selection and inadequate feature learning due to transforming features to reduce RFF features information. At the same time, a multisequence feature fusion algorithm is designed based on the input sequences, making full use of various sequence features and the single-channel and multichannel complementary features of each sequence feature, and adding a channel attention mechanism [17] and a temporal convolutional network (TCN) [18] to the network to redefine the channel feature weights and extract the temporal information embedded in the multisequence. The advantages of convolutional neural networks for fusion and extraction of multiple sequences features are fully exploited. To demonstrate the superiority of the proposed algorithm in this study, comparison simulations with other SEI algorithms are conducted, and the simulated results show that the SEI algorithm based on multiple sequence feature learning outperforms the benchmark algorithm at all signal-to-noise ratios. The comparison simulations of single sequence feature input with multiple sequence input and the controlled simulated groups of each module of the network are also conducted to further illustrate the rationality of the multi-feature fusion of the model in this study and the rationality of the construction of the multi-serial fusion convolutional network.

## Transmitter model and acquisition signal pre-processing

### Transmitter model

**Fig 1:**
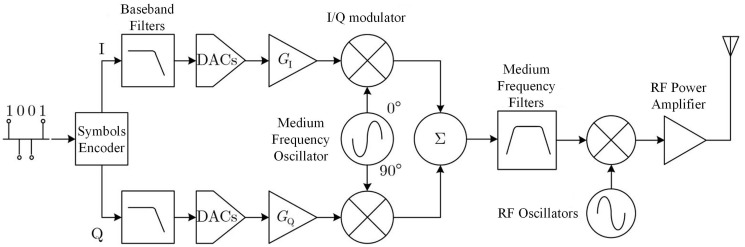
I/Q quadrature modulation transmitter.

The typical structure of an I/Q orthogonal modulation transmitter [19] is shown in Fig 1, which is basically identical to the structure of an actual communication transmitter used in practice. However, RFF mainly originates from uncontrollable or unintentional error factors in the design, manufacturing, and operation process of the transmitter, and these error factors are called distortion. Currently, it is generally believed that the possible sources of RFF in communication transmitters include but are not limited to distortion of I/Q modulators, filters, oscillators, and power amplifiers.

This section will summarize the mechanism of distortion in I/Q modulators, filters, oscillators, and power amplifiers, and provide corresponding distortion mathematical models.

Due to imperfections in the hardware production process, I/ Q modulators are subject to I/ Q imbalance, which is mainly manifested as gain mismatch, phase mismatch, and DC bias [20]. Assuming that *s*_*b*,*I*_(*t*) and *s*_*b*,*Q*_(*t*) are the baseband waveforms of the I/Q channels, respectively, the ideal baseband signal is

$$\begin{array}{c} s_{0}\left(t\right)=s_{b,I}\left(t\right)+js_{b,Q}\left(t\right) \end{array}$$ (1)

And the baseband signal carrying the I/Q modulator distortion can be expressed as

$$\begin{array}{c} s\left(t\right)=\left(1-g\right)\left(s_{b,I}\left(t\right)+c_{I}\right)+j\left(1+g\right)\left(s_{b,Q}\left(t\right)+c_{Q}\right)e^{j\phi } \end{array}$$ (2)

where *g* is the gain mismatch, *ϕ* is the phase deviation of the quadrature error, and *c*_*I*_ and *c*_*Q*_ are the DC components generated by the two mixers, respectively.

The filter distortion is mainly manifested by skewed amplitude-frequency response and fluctuations in ripple and group time delaybias [19]. Assuming that the ideal baseband shaping filter is *g*_*t*_, the ideal transmit signal is

$$\begin{array}{c} s_{0}\left(t\right)=e^{j(2\pi f_{c}t+\theta )}\sum_{k=1}^{L}\left(a_{k}g\left(t-kT_{0}-\tau \right)\right) \end{array}$$ (3)

where *f*_*c*_ is the carrier frequency, *θ* is the initial phase, *τ* is the time delay, *a*_*k*_ is the transmitted symbol sequence, and *T*_0_ is the symbol period. Let *G*_*f*_ be the frequency response of *g*_*t*_, then the frequency response of the distortion filter can be expressed as

$$\begin{array}{c} H\left(f\right)=G\left(f\right)A\left(f\right)e^{j\phi (f)} \end{array}$$ (4)

Where *A*_*f*_ = *a*_0_ + *a*_*n*_*cos*(2*πα*_*n*_*f*) denotes amplitude distortion, *ϕ*_*f*_ = *b*_0_ + *b*_*n*_*cos*(2*πβ*_*n*_*f*) is phase distortion, *a*_0_ and *b*_0_ are linear gain, *a*_*n*_ and *b*_*n*_ are fluctuating gain, and *α*_*n*_ and *β*_*n*_ depend on the period of amplitude ripple and delay fluctuation, then the signal carrying filter distortion can be expressed as

$$\begin{array}{c} s\left(t\right)=e^{j(2\pi f_{c}t+\theta )}\sum_{k=1}^{L}\left(a_{k}h\left(t-kT_{0}-\tau \right)\right) \end{array}$$ (5)

where $h_{t}=\int_{-\infty }^{\infty }H\left(f\right)e^{j2\pi ft}df$ is the distorted baseband shaping filter.

The oscillator distortion is mainly manifested as phase noise near the carrier frequency. For the ideal signal shaped as Eq (3), assuming that the phase noise is *φ*(*t*), the signal carrying the oscillator distortion can be expressed as

$$\begin{array}{c} s\left(t\right)=e^{j(2\pi f_{c}t+\theta +\varphi \left(t\right))}\sum_{k=1}^{L}\left(a_{k}g\left(t-kT_{0}-\tau \right)\right) \end{array}$$ (6)

*φ*(*t*) is equivalent to adding a time-varying additive factor to *f*(*c*). A 1st order autoregressive model is usually used to characterize *φ*(*t*)

$$\begin{array}{c} \varphi \left(t\right)=\left(1-c_{o}\right)\varphi \left(t-1\right)+c_{o}v\left(t\right) \end{array}$$ (7)

Where *φ*(0) = 0, *v*(*t*) is the Gaussian white noise with unit variance, and *c*(*o*) reflects the individual differences of transmitters. Eq (7) shows that: the larger the *c*_*o*_, the more random the *φ*(*t*) and the more obvious the perturbation to *f*_*c*_; conversely, the greater the correlation of *φ*(*t*), the higher the stability of the signal carrier frequency.

The effect of power amplifier distortion on the signal is mainly manifested in two ways: the amplitude/phase compression effect, where the signal amplitude is compressed in the saturated region, and the amplitude/phase conversion effect, where the signal amplitude produces additional phase in the nonsaturated region [19]. For narrowband amplifiers, the Taylor level model is usually used to describe them. It is assumed that the ideal signal input to the amplifier is

$$\begin{array}{c} s_{0}\left(t\right)=\rho \left(t\right)e^{j(2\pi f_{c}t+\theta )} \end{array}$$ (8)

Where *ρ*(*t*) = *s*_*b*,*I*_(*t*) + *js*_*b*,*Q*_(*t*) is the ideal complex baseband waveform, the signal carrying the amplifier distortion can be expressed as

$$\begin{array}{cc} s(t) & =\sum_{k=1}^{K}\lambda_{2k-1}{\left(s_{0}\left(t\right)\right)}^{k}{\left(s_{0}^{*}\left(t\right)\right)}^{k-1}\\ & =\lambda_{1}\rho \left(t\right)e^{j(2\pi f_{c}t+\theta )}+\sum_{k=2}^{K}\lambda_{2k-1}{\left|\rho \left(t\right)\right|}^{2k}\rho \left(t\right)e^{j(2\pi f_{c}t+\theta )} \end{array}$$ (9)

where λ_1_, λ_3_, …, λ_2*K*−1_ is the coefficient of the Taylor series and λ_1_ = 1. usually have λ_3_ < 0 and |λ_*k*_ < 0| decreases with increasing *k*. Therefore, the second term of Eq (9) is mainly characterized by λ_3_, which weakens the amplitude of the input signal and causes the AM/AM compression effect. When λ_*k*_ is complex, the signal amplitude is converted into additional phase, resulting in an AM/PM conversion effect.

The above distortion mathematical models to a certain extent demonstrate the process of actual RFF generation. The radiated source signals generated based on these distortion mathematical models contain RFFs simulated from the distortion mechanism of the radiated source, which is similar to the process of actual RFF generation in the radiated source. Therefore, the radiated source signals generated based on the above distortion mathematical models are similar to actual radiated source signals, which can be used as data for studying individual identification algorithms of radiated sources.

### Signal pre-processing

The pre-processing is divided into two main parts, the standardization processing and the generation of multiple sequence signals. Standardization processing means eliminating the interference of irrelevant factors without losing the signal information integrity. The standardization process includes symbol rate estimation, carrier synchronization, signal delay and phase estimation, interpolation filtering, and amplitude normalization. After the standardization process is completed, it is necessary to combine different signal sequence representations to convert the overharvest sequences into time domain I/Q sequences, time domain A/P sequences, and frequency domain A/P sequences after Fourier transform for this study.

The received signal after normalization is sampled at the sampling rate *F*_*s*_ to obtain a baseband signal complex sequence of length *N*

$$\begin{array}{c} r\left(n\right)=r_{I}\left(n\right)+jr_{Q}\left(n\right),n=0,\ldots ,N-1 \end{array}$$ (10)

The instantaneous amplitude *A*(*n*) and the instantaneous phase *P*(*n*) of the signal are defined as follows

$$\begin{array}{c} \left\{ \begin{array}{cc} A\left(n\right)=\sqrt{r_{I}^{2}\left(n\right)+r_{Q}^{2}\left(n\right)}, & \\ P\left(n\right)=\text{arctan}(\frac{r_{I}\left(n\right)}{r_{Q}\left(n\right)}, \end{array}\right. \end{array}$$ (11)

The Fourier transform can be used to obtain a frequency domain complex sequence as follows

$$\begin{array}{c} X\left(K\right)=X_{I}\left(K\right)+jX_{Q}\left(K\right)=DFT\left[r\left(n\right)\right]=\sum_{n=0}^{N-1}r\left(n\right)e^{-jk\frac{2\pi }{N}n},0\leq k\leq N-1 \end{array}$$ (12)

The signal amplitude spectrum *X*(*K*) and phase spectrum *F*(*K*) can be obtained by Φ(*K*), as shown in Eq (13).

$$\begin{array}{c} \left\{ \begin{array}{cc} F\left(K\right)=\sqrt{X_{I}^{2}\left(K\right)+X_{Q}^{2}\left(K\right)}, & \\ \Phi \left(K\right)=\text{arctan}(\frac{X_{I}\left(K\right)}{X_{Q}\left(K\right)}, \end{array}\right. \end{array}$$ (13)

A certain arrangement of the above time-domain sequence with the frequency-domain A/P sequence can yield the required multiple sequence signal representation in this study, as shown in Eq (14).

$$\begin{array}{c} {\left[\begin{array}{cccc} r_{I}\left(0\right) & r_{I}\left(1\right) & \ldots & r_{I}\left(N-1\right)\\ r_{Q}\left(0\right) & r_{Q}\left(1\right) & \ldots & r_{Q}\left(N-1\right)\\ A(0) & A(1) & \ldots & A(N-1)\\ P(0) & P(1) & \ldots & P(N-1)\\ F(0) & F(1) & \ldots & F(N-1)\\ \Phi (0) & \Phi (1) & \ldots & \Phi (N-1) \end{array}\right]}_{6\times N} \end{array}$$ (14)

## Multi-sequence fusion convolutional network design

**Fig 2:**
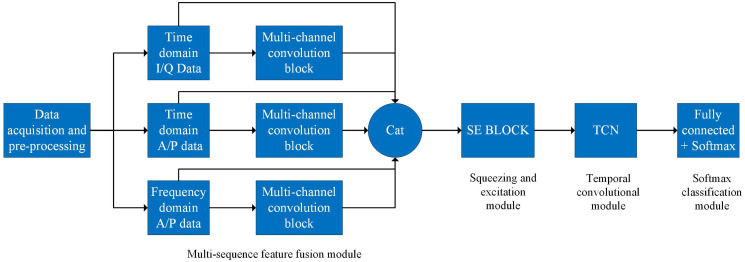
Structure of multi-sequence fusion convolutional network.

In order to more fully extract and efficiently identify the RFF features embedded in multiple sequences, this study constructs a multiple sequence fusion convolutional network structure, which can organically fuse and deeply mine the multiple sequence features of communication radiation sources. Under single-source conditions, the designed network architecture can accurately identify individuals by learning the unique characteristics of the radiation source. The network structure can capture subtle differences in the spectral data of radiation sources and use them to distinguish between different sources. Compared to traditional rule-based or feature engineering methods, this learning approach can better adapt to complex radiation source features and achieve higher recognition accuracy. As shown in Fig 2, the multi-sequence fusion convolutional network mainly contains four parts, which are multi-sequence feature fusion module, squeezing and excitation module, Temporal convolutional module and softmax classification module.

### Multi-sequence feature fusion module

The multi-sequence feature fusion module consists of three multi-channel convolutional blocks and a cancatenate layer. First, the RFF features contained in each of the time domain I/Q sequence, time domain A/P sequence, and frequency domain A/P sequence are extracted by the three multi-channel convolution blocks. The extracted feature sequences are then vector spliced with the original feature sequences in Cancatenate. The final output feature sequence has both deep and shallow features, both time domain and frequency domain features, with rich feature levels and rich content, which is more conducive to the subsequent feature learning and extraction.

**Fig 3:**
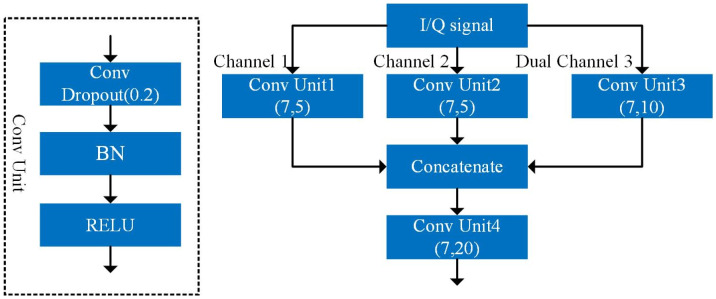
Multi-channel convolution block.

The structure of one of the multichannel convolution blocks is shown in Fig 3. The multichannel convolution block consists of three parallel convolution units (Conv Unit1, Conv Unit2, and Conv Unit3), a cancatenate layer (Cancatenate), and a convolution unit (Conv Unit4) composed of layers in turn. The convolutional unit consists of a 1D convolutional layer, a BN layer, and a ReLU activation function, which normalizes the data to mitigate the effect of gradient disappearance on the network, and a ReLU activation function that enhances the nonlinear representation of the network and mitigates the gradient disappearance problem. The dropout is set after the 1D convolutional layer to reduce the overfitting of the network during training by randomly subtracting some neurons.

The multi-channel convolution block shunts a certain set of received two-channel sequence features into two single channels, channel 1 and channel 2, and one double channel, channel 3. Then, these three input data streams are fed to Conv Unit1, Conv Unit2, and Conv Unit3, respectively, and the sequence features single and dual channel features are learned. Subsequently, the learned multi-channel features are fused in Cancatenate and fed to Conv Unit4 for further fusion and extraction of the complementary features of single and dual channels.

### Squeeze and excitation block (SE block)

**Fig 4:**
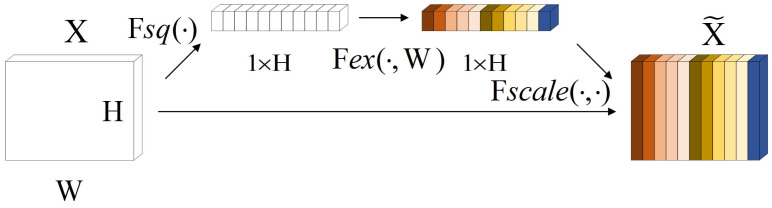
Modified SE block structure.

The Attention Mechanism [21] can help the model assign different weights to each part of the input feature vector to extract more critical and important information, so that the model can make more accurate judgments without imposing greater overhead on the model’s computation and storage. Squeeze and incentive network (SENet) [17] is a channel attention mechanism, and the squeeze and incentive block (SE block) structure is the core structure in SENet. In this study, the inputs and outputs of the SE block structure are slightly modified, and its structure is shown in Fig 4. For the sequence feature *X* (*X* ∈ *R*^*W*×*H*^) output from the upper layer of the neural network, the channel statistics vector *Z* (*Z* ∈ *R*^1×*H*^) can be generated by global average pooling *F*_*sp*_, where the *i*th element of *Z* is calculated by the following equation.

$$\begin{array}{c} Z_{i}=F_{sp}\left(X_{i}\right)=\frac{1}{W}\sum_{j=1}^{W}X_{i}\left(1,j\right) \end{array}$$ (15)

*Z* can generate the weight vector *S* of the sequence feature *X* channel by a specific variation *F*_*ex*_ as shown in Eq (16). where *δ* refers to the *RELU* function, *σ* is the *Sigmoid* activation function, $W_{1}\in R^{\frac{H}{r}\times H}$, $W_{2}\in H\times R^{\frac{H}{r}}$.

$$\begin{array}{c} S=F_{ex}\left(Z,W\right)=\sigma \left(g\left(Z,W\right)\right)=\sigma \left(W_{2}\delta \left(W_{1}Z\right)\right) \end{array}$$ (16)

After obtaining the weight vector *S* for the channel of sequence feature *X*, the channel feature of *X* can be rescaled by multiplication using *S* to generate a new feature vector $\tilde{X}$ ($\tilde{X}\in R^{W\times H}$). the *i*th element of $\tilde{X}$ is calculated by Eq (17), where $\tilde{X}=\left[\tilde{s_{1}},\tilde{s_{2}},\ldots ,\tilde{s_{H}}\right]$.

$$\begin{array}{c} \tilde{s_{i}}=F_{scale}\left(x_{i},s_{i}\right)=s_{i}x_{i} \end{array}$$ (17)

**Fig 5:**
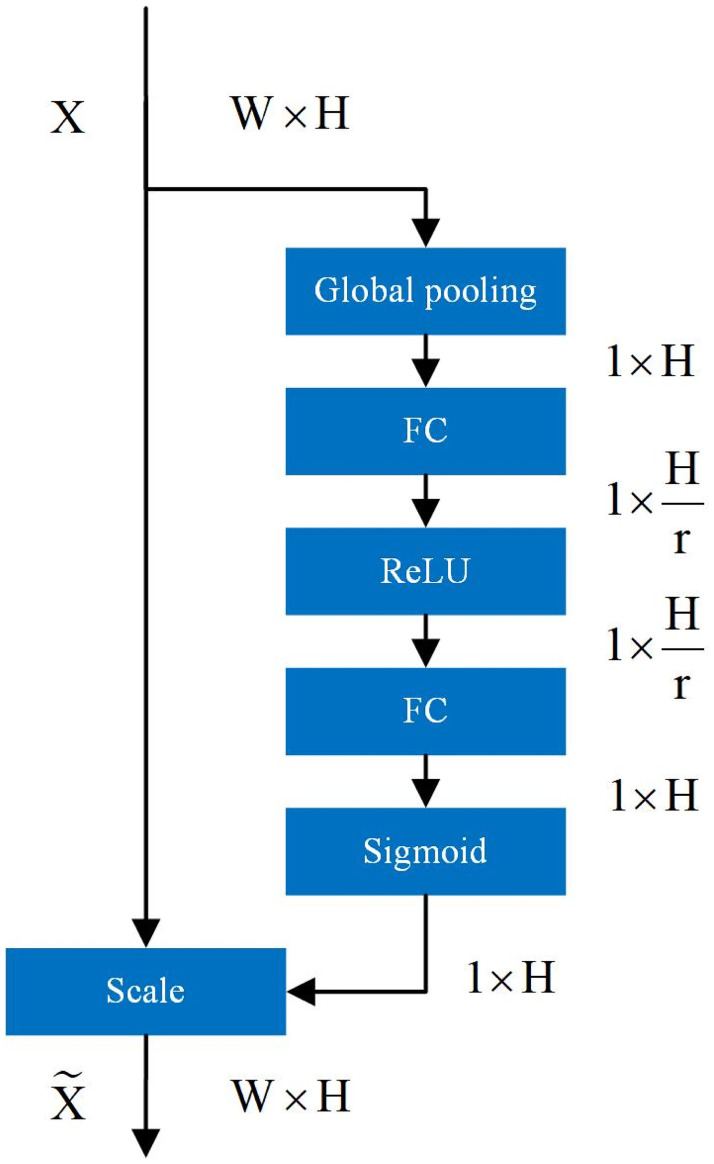
Inserted SE block structure.

The specific structure of the SE block inserted in this study is shown in Fig 5.

### Temporal convolutional module and softmax classification module

TCNs use convolution instead of recursion for modeling time series and have the advantage of being able to extract an effective representation of the input data by building multiple filters. The main structures used in TCN models are causal convolution and inflationary convolution, and connections are established by constructing blocks of temporal residuals.

Causal convolution ensures that there is no future information leakage, and the convolution operation is performed in strict chronological order, i.e., the convolution operation at moment *t* occurs only on the data before the moment *t* − 1 and *t* − 1 in the previous layer, so that the convolution kernel is *F* = (*f*_1_, *f*_2_, …, *f*_*K*_), where *K* is the size of the convolution kernel, and the input sequence *X* = (*x*_1_, *x*_2_, …, *x*_*T*_), then the causal convolution at *x*_*T*_ is

$$\begin{array}{c} F\left(x_{T}\right)=\sum_{k=1}^{K}f_{k}x_{T-K+k} \end{array}$$ (18)

To address the modeling problem of traditional convolutional neural networks, which require linear stacking of multiple layers of convolution to fit longer time series, TCNs reduce the number of convolutional layers by increasing the range of perceptual fields per layer using inflation convolution. The difference between inflationary convolution and normal convolution is that inflationary convolution adds spacing to the standard convolutional kernel, thus expanding the contact area of the model.

Let the filter be *F* = (*f*_0_, *f*_1_, …, *f*_*k*−1_), the sequence signal be *S* = (*s*_0_, *s*_1_, …, *s*_*T*_), and the value *s*_*t*_ at the moment of *t* in the input sequence is convolved with the expansion to obtain

$$\begin{array}{c} F\left(s_{t}\right)=\left(F_{d*S}\right)s_{t}=\sum_{i=0}^{I-1}f_{i}s_{t-d}i \end{array}$$ (19)

where: *d* is the expansion factor; *I* is the filter size. Thus the operation of the expansion convolution is equivalent to introducing a fixed interval between two adjacent filters, which increases the perceptual field range.

The TCN model uses a residual connection to solve the model training degradation problem, by learning the amount of residuals between the input to output target function and the original function, and adding the residuals to the original input to obtain the final target mapping function, if the input variable is *x*_*l*_ and the actual mapping of the target output is *H*(*x*_*l*_), the residual mapping is

$$\begin{array}{c} F\left(x_{l},W_{l}\right)=H\left(x_{l}\right)-x_{l} \end{array}$$ (20)

where *x*_*l*_ is the input of the *l*th layer; *W*_*l*_ is the weight matrix of the *l*th layer. The input *x*_*l*_ is passed directly to the output as the initial result by means of a shortcut connection (shortcut), and the output result is *H*(*x*_*l*_) = *F*(*x*_*l*_, *W*_*l*_) + *x*_*l*_, when *F*(*x*_*l*_, *W*_*l*_), *H*(*x*_*l*_) = *x*_*l*_.

**Fig 6:**
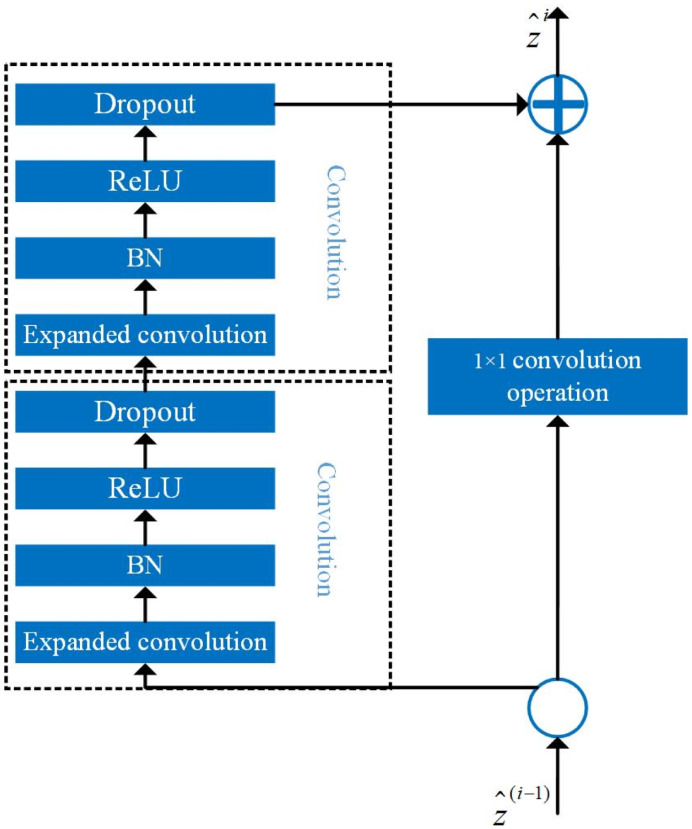
TCN residual block.

A typical TCN consists of a TCN residual block (TRB) stack. The structure of TRB is shown in Fig 6, containing convolutional layers and nonlinear mappings, and the main parameters are the convolutional kernel size and the expansion factor.

The convolutional layer performs an inflated convolution operation between the feature vector of the upper layer and the convolutional kernel of the current layer, where the input vector of the upper layer is ${\tilde{z}}^{i-1}=\left({\tilde{z}}_{1}^{i-1},{\tilde{z}}_{2}^{i-1},\ldots ,{\tilde{z}}_{T}^{i-1}\right)$ and the output of the current layer to the lower layer vector is ${\tilde{z}}^{i}=\left({\tilde{z}}_{1}^{i},{\tilde{z}}_{2}^{i},\ldots ,{\tilde{z}}_{T}^{i}\right)$, and ReLU is used as the activation function between the convolutional layers to enhance the characteristics of the original signal and reduce noise. Afterwards, the neural network training unit is deactivated using a random deactivation (Dropout) layer, which allows the network’s generalization performance to be improved, randomly responding to the nodes of the network, ensuring the sparsity of the network and reducing overfitting [22].

**Fig 7:**
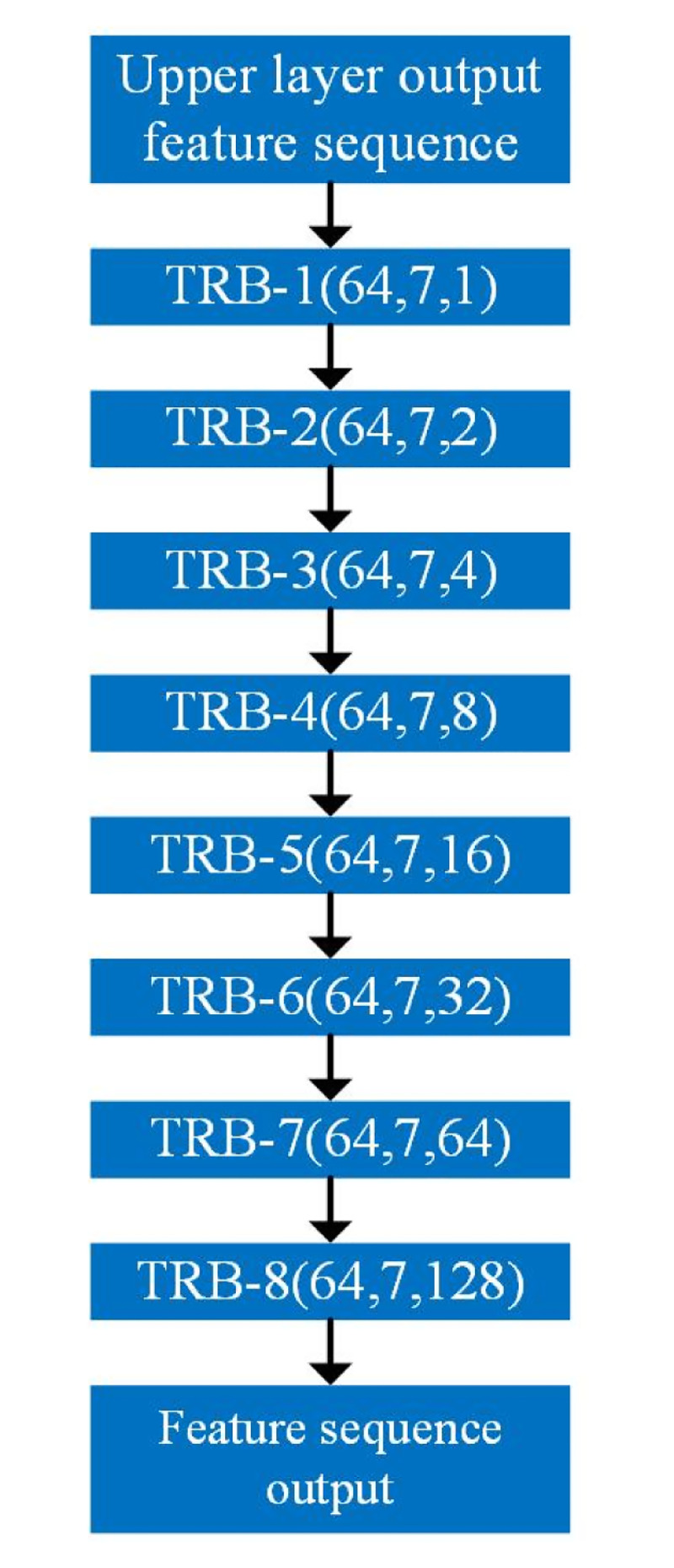
Temporal convolutional module.

In this study, an improved TCN feature extraction model is constructed by connecting the temporal residual blocks and using residual hopping and inflated convolution inside the residual blocks to maximize the utilization of forward resources and reduce the network degradation. The temporal convolution module is shown in Fig 7, which consists of eight temporal residual blocks stacked sequentially, with the number of convolution kernels being 64, the size of convolution kernels being 7, and the expansion coefficients being 1,2,4,8,16,32,64,128. Each residual block has the same expansion coefficients, and the expansion coefficients grow layer by layer between different residual blocks to ensure that the local perceptual fields of the convolution can be expanded in a sequential manner.

The temporal convolution module is followed by the softmax classification module. softmax classification module mainly consists of one layer of full convolutional layers. Let the output of the temporal convolution module be *out*_64×*n*_ = [*out*_1_, *out*_2_, …, *out*_*n*_], where *out*_*n*_ contains enough feature information. Therefore, the input received by the fully-connected layer is *out*_*n*_, the number of neurons in the fully-connected layer is the number of target categories, and the activation function uses the *Softmax* function.

## Simulated background

### Radiated source signal parameters

To verify the performance of the proposed algorithm, this study generates radiated source data using the transmitter distortion mathematical model mentioned earlier. The relevant parameters of the dataset are set as follows:

- The number of radiated sources and distortion parameters: This study summarized 9 distortion parameters, namely *g*, *ϕ*, *c*_*I*_, *c*_*Q*_, *a*_*n*_, *b*_*n*_, *c*_*o*_, λ_3_, and λ_5_, as the fingerprint features controlling the radiation source, using a mathematical model of transmitter distortion. In the simulation process, a specific set of values for the 9 distortion parameters represents a specific radiation source. The study employed 7 different sets of distortion parameter values, resulting in the simulation of 7 radiation sources with distinct fingerprint features. There are 7 radiated sources with a default distortion parameter of {*a*_0_, *b*_0_, *α*_*n*_, *β*_*n*_} = {1, 0, 4, 4}. The distortion parameters of the other radiated sources are shown in Table 1 [20].
- Transmitted signal information: Random sequences are generated through a random number function for simulation, to eliminate the effect of signal content on individual identification of radiated sources.
- The method of signal waveform shaping: A raised cosine shaping filter with a roll-off factor of 0.35 is used to shape the baseband signal waveform.
- Signal frequency: The transmission frequency is 1750 kHz, and the signal is sampled and demodulated to zero intermediate frequency during signal reception.
- Modulation parameters: QPSK modulation mode, modulation rate is 500 kBaud, symbol number L = 200.
- Sampling rate: 10 MHz, therefore 20 sampling points per symbol.
- The form of the signal after receiving and processing: Zero-intermediate frequency complex baseband signal.

**Fig 8:**
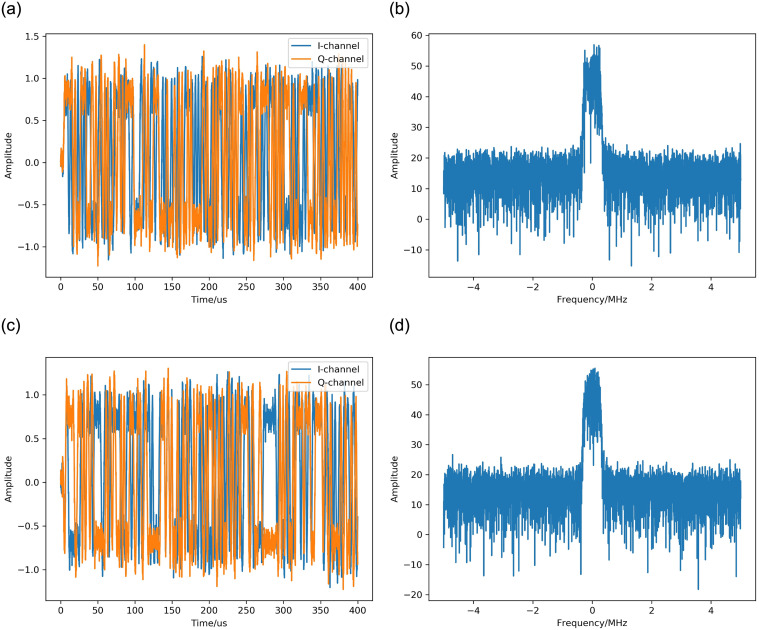
The waveform and spectrum of the simulated signal. (a) The waveform of the signal received under condition 1. (b) The spectrum of the signal received under condition 1. (c) The waveform of the signal received under condition 2. (d) The spectrum of the signal received under condition 2.

According to the above simulation settings, the time-domain waveform and frequency-domain spectrum of the signal can be obtained, as shown in Fig 8.

**Table 1:** Aberration parameters of different radiation sources. (In each condition, only one radiation source is active. For example, Condition 3 indicates that only the third radiation source is active).

Parameters	Condition 1	Condition 2	Condition 3	Condition 4	Condition 5	Condition 6	Condition 7
*g*	0.0299	0.0188	0.0081	-0.0025	-0.0128	-0.023	-0.0329
*ϕ*	0.0137	0.0093	0.005	0.0006	-0.0038	-0.0081	-0.0125
*c*_*I*_	0.0142	0.0097	0.0052	0.0007	-0.0038	-0.0083	-0.0128
*c*_*Q*_	0.0147	0.0102	0.0057	0.0012	-0.0033	-0.0078	-0.0123
*a*_*n*_	-0.064	-0.0429	-0.0218	-0.0007	0.0204	0.0415	0.0627
*b*_*n*_	-0.074	-0.0498	-0.0256	-0.0014	0.0228	0.047	0.0713
*c*_*o*_	0.0002	0.001	0.0018	0.0026	0.0034	0.0042	0.005
λ_3_	-0.2915-0.0079i	-0.0003-0.0004i	-0.4371-0.0092i	-0.1459-0.0066i	-0.5827-0.0096i	-0.0731-0.0042i	-0.3643-0.0085i
λ_5_	0.0295+0.0005i	0.0001+0.0004i	0.0821+0.0048i	0.0338+0.0014i	0.0537+0.0029i	0.0571+0.0035i	0.0484+0.0022i

### Training and testing sample configuration

Each radiation source is simulated to generate 6400 signal samples during training, and Gaussian white noise is added to these 6400 samples, and the signal-to-noise ratios are equally distributed from 0dB to 30dB at 2dB intervals, i.e., there are 400 radiation source samples under each signal-to-noise ratio (SNR) in each class. At the same time, 300 samples are randomly selected for training and updating the network parameters for each of the 400 signal-to-noise samples in each class, and the remaining 100 are used for validation.

When testing, the corresponding Gaussian white noise, modulation rate offset and other effects are added. The specific parameters are as follows: Gaussian white noise ranges from 0dB to 30dB, and in 2dB intervals. The modulation rate offset values are 0 kBaud to 150 kBaud, and 10 kBaud intervals. Also 800 samples are generated for each S/N ratio under each type of radiation source, and the modulation rate effect is added at a modulation rate offset value for every 50 samples of the 800 samples.

### Implementation details

In this study, four simulations are set up to evaluate the algorithms proposed in this study. In the first simulation the algorithm of this study is compared with four algorithms, which are vector map based specific radiation source identification algorithm [13], VMD based communication radiation source individual identification algorithm [14], multi-domain feature fusion based communication radiation source individual identification algorithm [15], and multi-feature fusion classification algorithm for communication specific radiation source identification [16]. The second simulation explores the effect of signal modulation rate variation on network identification performance. The third simulation explores the network performance of the proposed network under different signal sequences, which are classified into four types, namely, time-domain I/Q sequences, time-domain amplitude phase sequences, frequency-domain amplitude phase sequences and their fusion sequences. In the fourth simulation we build three sets of controlled frameworks, MODEL-A (multi-sequence feature fusion module removed), MODEL-B (squeezing and excitation module removed), and MODEL-C (temporal convolutional module removed).

The structure and parameters of the multi-sequence fusion convolutional network in the simulations of this study are shown in Table 2. Combined with the description of the network structure details in the previous section, the specific structure and parameters of the multi-sequence fusion convolutional network in the simulations of this paper can be obtained.

**Table 2:** Multi-sequence fusion convolutional network structure and parameters.

Network Structure			Parameters	Input	Output
**multi-sequence feature fusion module**	Multi-channel convolution block 1	Convolution unit 1	(7,5)	2000×2	2000×5
		Convolution unit 2	(7,5)	2000×2	2000×5
		Convolution unit 3	(7,10)	2000×2	2000×10
		Cancatenate 1	(7,10)	2000×5,2000×5,2000×10	2000×20
		Convolution unit 4	(7,20)	2000×20	2000×20
	Multi-channel convolution block 2			2000×2,2000×2,2000×2	2000×20
	Multi-channel convolution block 3			2000×2,2000×2,2000×2	2000×20
	Cancatenate			2000×20,2000×20,2000×20 2000×2,2000×2,2000×2	2000×66
**squeezing and excitation module**			(r = 4)	2000×66	2000×66
**Temporal convolutional module**	TRB-1		(64,7,1)	2000×66	2000×64
	TRB-2		(64,7,2)	2000×64	2000×64
	TRB-3		(64,7,4)	2000×64	2000×64
	TRB-4		(64,7,8)	2000×64	2000×64
	TRB-5		(64,7,16)	2000×64	2000×64
	TRB-6		(64,7,32)	2000×64	2000×64
	TRB-7		(64,7,64)	2000×64	2000×64
	TRB-8		(64,7,128)	2000×64	1×64
**Softmax classification module**	FCN		(64,7)	1×64	1×7

All algorithms use NVIDIA Quadro RTX 6000 and Keras2.6.0 Tensorflow-GPU2.4.0 as the back-end simulated platform to facilitate performance comparisons between networks. Uniform hyperparameters were established for network training and testing. adam optimizer was used to optimize the network in the simulations. The initial learning rate was set to 0.001, the absolute cross entropy was the loss function, and the gradient update batch size was 64. during the training process for the validation of the loss assessment set, when the validation loss still did not improve after 30 epochs, we stopped the training process to save the weights of the model with the minimum validation loss. Set the maximum number of training hours of the algorithm on the training set to 200. first construct the test model, then load the trained weights file, and finally predict the radiation source class for each test signal.

## Simulated results and discussion

### Performance comparison between this algorithm and other algorithms

Firstly, the performance performance of the algorithm in this study is tested. Two single feature extraction algorithms [13, 14] and two multi-feature extraction algorithms [15, 16] are selected for comparison with the algorithm in this study. The literature [13] uses only vector map to extract features for recognition and is noted as vector map algorithm. The literature [14] uses only the high-frequency component of the variational modal decomposition to extract features for recognition, and is noted as the variational modal decomposition algorithm. Similar to the algorithm in this study, literature [15] and literature [16] also use the idea of multi-feature extraction, and literature [15] uses multi-threshold data such as time-domain I/Q sequences, power spectrum sequences and integrated bispectral sequences of I/Q two-way to extract features for recognition, which is noted as multi-domain feature fusion algorithm. The literature [16] uses the fusion of wavelet eigencoefficient matrix 2D projection image, bispectral eigencoefficient matrix 2D projection image, and Hebert sign coefficient matrix 2D projection image to extract features for recognition, which is noted as multi-projection feature fusion algorithm.

**Fig 9:**
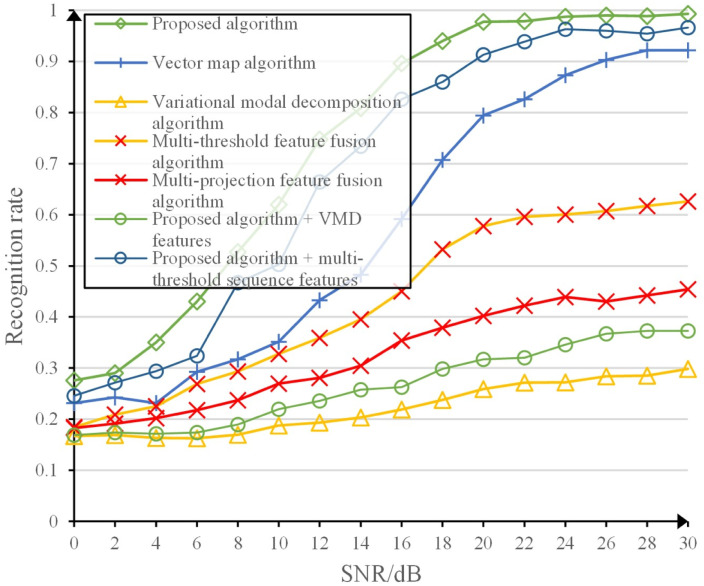
Recognition rate comparison curve.

Table 3 and Fig 9 show the average recognition accuracy of the algorithm in this study and the comparison algorithm under the corresponding inputs, as well as the graph of the recognition rate with the variation of signal-to-noise ratio. It can be seen that the algorithm in this study has the highest final recognition rate and outperforms the other algorithms in terms of recognition accuracy and noise immunity performance, and the average recognition rate is ahead by about 17%. The vector map algorithm and the variational modal decomposition algorithm are both single feature extraction algorithms, and the recognition effect differs greatly, indicating that the selection of features has a greater impact on the recognition rate, and the vector map features are better than the sequence features of the variational modal decomposition. The multi-domain feature fusion algorithm and the multi-projection feature fusion algorithm are both multi-feature extraction algorithms, and the recognition effect of the multi-domain feature fusion algorithm is better than that of the multi-projection feature fusion algorithm. The main reason is that the basic features in the multi-domain feature fusion algorithm contain time domain I/Q sequences and more fingerprint features of the radiation source, however, the basic features of the multi-projection feature fusion algorithm are the projection image features generated by multiple transformations, and the intermediate process loses a lot of fingerprint features of the radiation source to cause the algorithm recognition effect is not good. The recognition effect of the proposed network in this study is better than that of the variational modal decomposition algorithm and the multi-domain feature fusion algorithm in the case of the same input features, respectively. This is because the network used in the comparison algorithm does not construct a network according to the characteristics of the input signal, and simply splices the network for recognition, which leads to poor recognition effect. The multi-sequence fusion convolutional network constructed in this study fully exploits the fingerprint features contained in the input features. The algorithm in this study performs very well with the input of the multiple sequence features proposed in this study.

**Table 3:** Comparison of average recognition accuracy of different SEI algorithms under different input features.

SEI algorithm	Sampling signal form	Number of sample points utilized	Input feature form	Feature size	Average accuracy rate
**Proposed algorithm**	4000 points of baseband complex sequence signal sampling points	2000	Time domain I/Q sequences Time domain A/P sequence Frequency domain A/P sequences	6×2000	0.737
**Vector map algorithm**		4000	Grayscale vector image	300×300×3	0.570
**Variational modal decomposition algorithm**		4000	Second eigenmode function signal sequence	1×4000	0.222
**Multi-domain feature fusion algorithm**		4000	Time domain I/Q sequences Power Spectrum Sequence I-way integral bispectral sequence Q-way integral bispectral sequence	5×1280	0.429
**Multi-projection feature fusion algorithm**		4000	Two-dimensional projection of wavelet eigencoefficient matrix Two-dimensional projection of the bispectral eigencoefficient matrix Two-dimensional projection of the Hebert sign coefficient matrix	224×224×3 224×224×3 224×224×3	0.326
**Proposed algorithm**		4000	Second eigenmode function signal sequence	1×4000	0.267
**Proposed algorithm**		4000	Time domain I/Q sequences Power Spectrum Sequence I-way integral bispectral sequence Q-way integral bispectral sequence	5×1280	0.680

**Fig 10:**
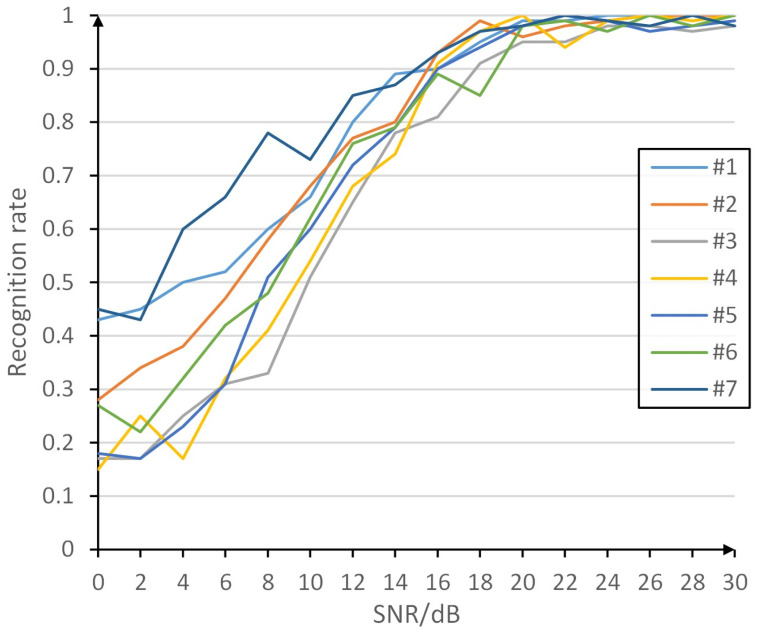
Variation of recognition rate of each category with different SNR.

**Fig 11:**
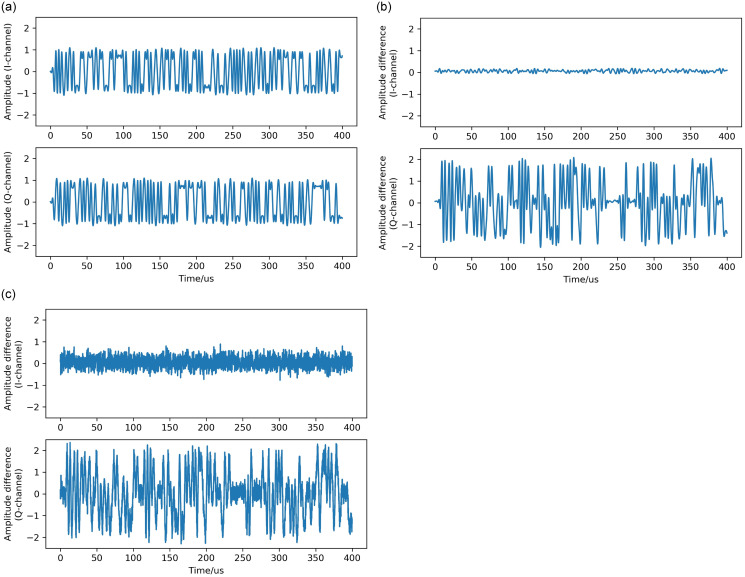
Schematic diagram of the effect of Gaussian white noise on RFFs. (a) Signal waveform of the ideal received signal. (b) Difference between the received signal with transmitter 1 distortion and the ideal received signal. (c) Difference between the received signal with transmitter 1 distortion and 10dB noise and the ideal received signal.

Fig 10 demonstrates the variation of the recognition rate of each type of radiation source at different signal-to-noise ratios, where the recognition is generally poor at low signal-to-noise ratios and generally good at high signal-to-noise ratios. As shown in Fig 11, which demonstrates the schematic diagram of the effect of Gaussian white noise on RFFs, Fig 11(a) is the I/Q two-way signal waveform diagram of the ideal received signal without transmitter distortion, which represents the ideal case without transmitter distortion. Fig 11(b) shows the difference between the received signal without noise but containing the effect of transmitter 1 distortion and the ideal signal, which is mainly affected by the transmitter 1 distortion and contains most of the transmitter 1 fingerprint information. Fig 11(c) shows the difference between the received signal containing noise and the ideal signal with the influence of transmitter 1 distortion. Compared with Fig 11(b), it can be seen that the Gaussian white noise affects the difference between the received signal with the influence of transmitter 1 distortion and the ideal signal, which disturbs the fingerprint information of transmitter 1 to a certain extent and increases the difficulty of extracting and identifying the fingerprint information of transmitter 1. Therefore, under the low signal-to-noise ratio, the greater the influence of Gaussian white noise, the more difficult the extraction and identification of fingerprint information, and the lower the identification rate of the radiation source.

### The effect of modulation rate offset on recognition performance is divided

**Fig 12:**
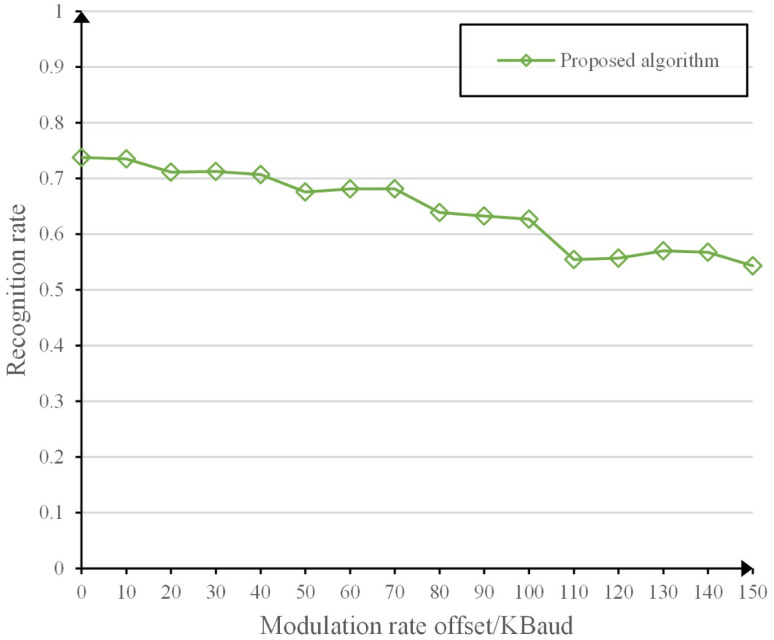
Average recognition rate at different modulation rate offsets.

Simulations are conducted to investigate the effect of modulation rate parameter variations on the identification performance of the proposed algorithm. Based on the data obtained from the simulations, we find the average recognition rate of the radiation source for the algorithm in this paper with different modulation rate offsets. Fig 12 shows that when the modulation deviation is below 100 kBaud, the proposed algorithm shows a higher recognition rate, which is higher than the average recognition rate of the benchmark algorithm without modulation rate offset. When the modulation deviation is between 100 kBaud and 150 kBaud, the average recognition rate of this algorithm still remains above 50%, which indicates that this algorithm has good robustness. In order to further improve the robustness of this paper, we can add samples of multiple modulation rate signals when constructing the training set to improve the adaptability of this paper’s algorithm to modulation deviations.

### Classification recognition rate for different sequence feature inputs

**Fig 13:**
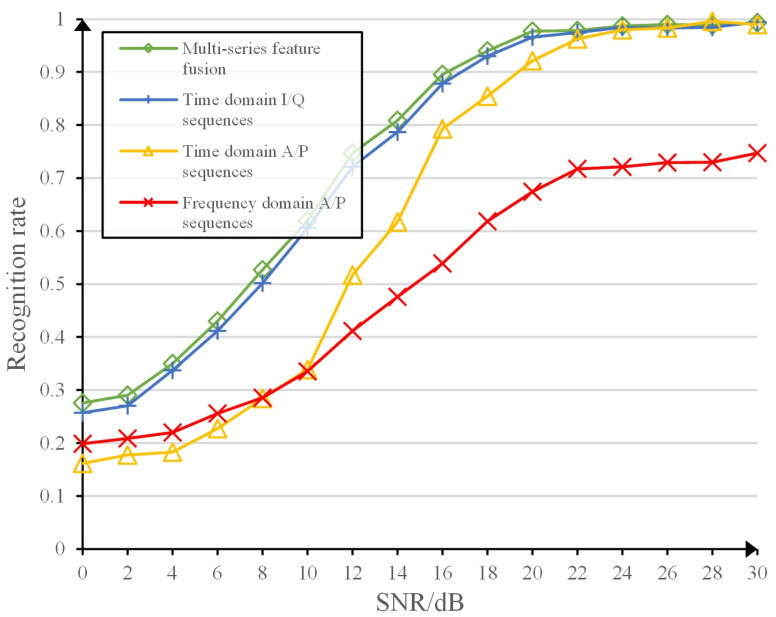
Algorithm performance comparison of single sequence features and multiple input sequence features.

The recognition results of the multi-sequence feature fusion convolutional neural network-based approach are compared with those of the single sequence feature-based neural network approach. The recognition results of the network with different signal-to-noise ratios are obtained as shown in Fig 13. The analysis of the simulated results shows that the algorithm with fusion of multiple sequence features is better than the algorithm with single sequence features, especially at lower signal-to-noise ratios. Among the algorithms based on single sequence features, the algorithm based on time domain sequence is significantly better than the algorithm based on frequency domain sequence, which indicates that the time domain sequence contains more information about the individual fingerprint of the radiation source, while the frequency domain sequence has undergone Fourier transform, which to a certain extent loses or transforms the fingerprint features of the radiation source, making it more difficult to mine the fingerprint features. The algorithm based on time domain I/Q sequences is better than the algorithm based on time domain AP sequences, which indicates that the time domain I/Q sequences contain more and more essential information of individual fingerprints of radiation sources. Therefore, the algorithm based on multi-domain feature fusion outperforms the algorithm with single-domain features in recognition results, especially at low signal-to-noise ratio.

### Comparison of the functions of each module

**Fig 14:**
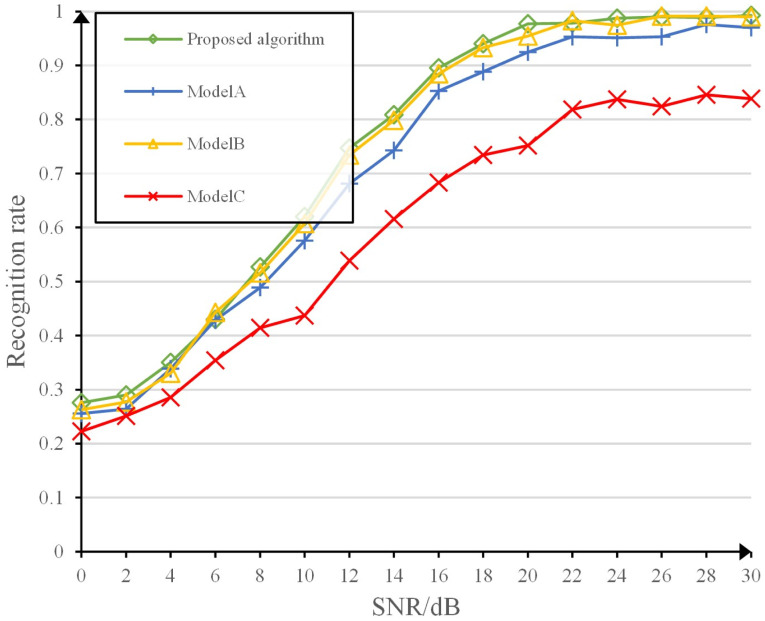
Performance comparison between modules.

The recognition accuracy of the model in this study is compared with ModelA, ModelB, and ModelC. Fig 14 shows that the proposed model provides the best recognition performance, which indicates that the advantages of the modules are complementary and their combination leads to a superior model. the recognition performance of ModelC is significantly degraded, indicating the importance of temporal correlation feature extraction of the input data as well as global feature extraction.

## Conclusion

In this study, a new efficient radiation source individual recognition algorithm with multiple sequence inputs by adding attention mechanism is proposed for extracting features from multiple sequence representations of signals. Simulation results show that the algorithm has the advantages of high recognition accuracy, good robustness and stability. It is also shown that using an efficient model structure and extracting features from spatial and temporal perspectives has great potential in communication systems.
